# Genome-Wide Analysis Reveals a Major Role in Cell Fate Maintenance and an Unexpected Role in Endoreduplication for the *Drosophila* FoxA Gene *Fork Head*

**DOI:** 10.1371/journal.pone.0020901

**Published:** 2011-06-16

**Authors:** Rika Maruyama, Elizabeth Grevengoed, Peter Stempniewicz, Deborah J. Andrew

**Affiliations:** Department of Cell Biology, The Johns Hopkins University, School of Medicine, Baltimore, Maryland, United States of America; Stockholm University, Sweden

## Abstract

Transcription factors drive organogenesis, from the initiation of cell fate decisions to the maintenance and implementation of these decisions. The *Drosophila* embryonic salivary gland provides an excellent platform for unraveling the underlying transcriptional networks of organ development because *Drosophila* is relatively unencumbered by significant genetic redundancy. The highly conserved FoxA family transcription factors are essential for various aspects of organogenesis in all animals that have been studied. Here, we explore the role of the single *Drosophila* FoxA protein Fork head (Fkh) in salivary gland organogenesis using two genome-wide strategies. A large-scale in situ hybridization analysis reveals a major role for Fkh in maintaining the salivary gland fate decision and controlling salivary gland physiological activity, in addition to its previously known roles in morphogenesis and survival. The majority of salivary gland genes (59%) are affected by *fkh* loss, mainly at later stages of salivary gland development. We show that global expression of Fkh cannot drive ectopic salivary gland formation. Thus, unlike the worm FoxA protein PHA-4, Fkh does not function to specify cell fate. In addition, Fkh only indirectly regulates many salivary gland genes, which is also distinct from the role of PHA-4 in organogenesis. Our microarray analyses reveal unexpected roles for Fkh in blocking terminal differentiation and in endoreduplication in the salivary gland and in other Fkh-expressing embryonic tissues. Overall, this study demonstrates an important role for Fkh in determining how an organ preserves its identity throughout development and provides an alternative paradigm for how FoxA proteins function in organogenesis.

## Introduction

The Fox family of winged helix DNA binding transcription factors is quite large, with more than 40 known family members in mammals and 18 in flies [1], [2] (R.M. and D.J.A., unpubl.). The mammalian proteins have wide-ranging activities from controlling development and differentiation of dopaminergic neurons to regulating the acquisition of vocal learning [2], [3]. During development, Fox family members regulate specialization of endothelial cells, formation of the lymphatic vessels, and development of the cardiac outflow tract [4]. Other Fox family members regulate melanocyte differentiation, skin pigmentation, and the development of specialized skin appendages, such as hair and nails [5], [6]. Fox proteins also play key roles in the immune system, regulating thymus development, affecting immune suppression and autoimmunity [7]. In addition, Fox proteins regulate such basic functions as cell growth and proliferation, and contribute to both tumor development and metastasis [8].

The FoxA1-3 proteins, originally known as hepatic nuclear factors (HNF-3α, β and γ), are among the best-studied members of the Fox protein family [9]. FoxA proteins are required for the development of the liver, lungs, pancreas and midbrain dopaminergic neurons [3], [10], [11], [12]. In several organs, including those that derive from the gut endoderm, the *foxA1* and *foxA2* genes appear to have largely overlapping functions. The late endodermal loss of each gene alone has very little affect, whereas the simultaneous loss of both genes results in a complete failure of internal organs, such as the liver, to form [9]. In other contexts, FoxA function is not redundant. In addition to its overlapping expression with *foxA1* in the definitive endoderm and multiple endodermal derivatives, *foxA2* is expressed earlier in the node and anterior primitive streak [13], [14], [15]. The complete knockout of *foxA2* results in the absence of the notochord, failure to form a gut tube and defects in derivatives from multiple germ layers [16], [17]. Unlike most other transcription factors, the FoxA1 protein has been shown to bind and open chromatin, suggesting that it functions as a “pioneer” protein providing target gene access for other tissue-specific transcription factors [18]; recent studies, however, showing FoxA1 also binds DNA in a relatively closed chromatin conformation challenge this model [19].

Insight into the function and activities of the FoxA genes has also come from studies of model organisms in which redundancy is less of an issue; the worm *C. elegans* and the fruitfly *D. melanogaster* each encode only a single FoxA homologue, PHA-4 and Fork head (Fkh) [20], [21], [22]. Microarray analysis comparing gene expression profiles of worms containing extra pharyngeal cells to those without pharyngeal cells, revealed a large number of pharyngeal expressed genes with consensus binding sites for the worm FoxA protein PHA-4 [23], which is essential for pharyngeal development [20], [21]. Analysis of several pharyngeal gene enhancers suggested a model wherein PHA-4 directly activates expression of most or even all pharyngeal-specific genes [23]. The model further suggested that PHA-4 pharyngeal target genes with high affinity binding sites are activated early when PHA-4 concentrations are low, whereas other targets with low affinity binding sites are activated only at late stages when levels of PHA-4 are sufficiently high.

**Figure 1:**
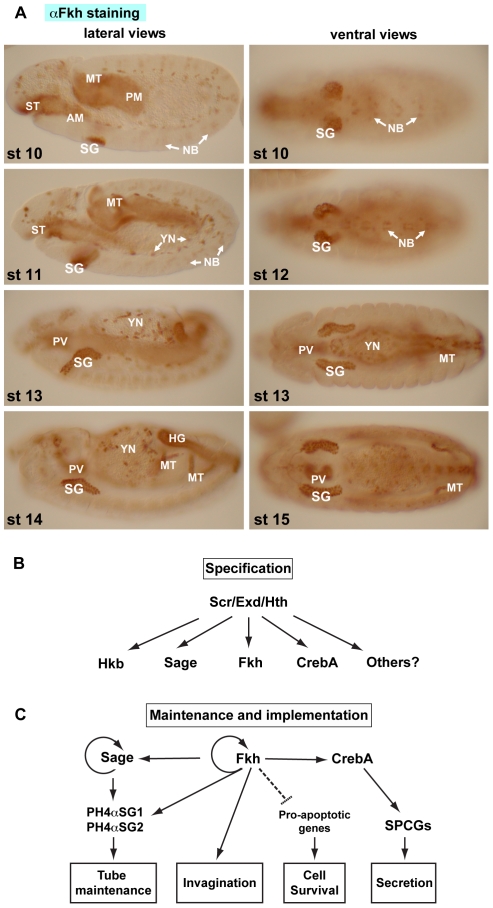
Fkh plays a major role in salivary gland development. (A) Fork head (Fkh) is expressed in the secretory cells of the salivary gland (SG), anterior and posterior midgut primordia (AM and PM), stomadeum (ST), Malpighian tubules (MT), a subset of neuroblasts (NB), yolk nuclei (YN), hindgut (HG) and proventriculus (PV). (B) SG specification requires parasegment 2-expressed Sex combs reduced (Scr) and two more widely-expressed cofactors, Extradenticle (Exd) and Homothorax (Hth). Scr with Exd and Hth activate several early SG transcription factors, including Huckebein (Hkb), Sage, Fkh, CrebA and others. Expression of Scr and Hth and nuclear Exd localization disappear during embryonic stage 11. Thus, Scr, Exd and Hth are not involved in maintaining SG gene expression. Some early-expressed transcription factors, such as Hkb, are also only transiently expressed in the SG. (C) Three early expressed transcription factors, Sage, Fkh and CrebA, continue to be expressed in the SG until early pupal stages when the cells are histolyzed during metamorphosis. Maintained SG expression of these genes requires Fkh, which directly regulates its own expression and that of both CrebA and Sage. Fkh is required for SG invagination and SG cell survival. Fkh and Sage control expression *PH4αSG1* and *PH4αSG2*, two genes required to maintain an open patent SG lumen. CrebA elevates expression of secretory pathway component genes (SPCGs) required for the high level secretory capacity of the SG.

In flies, studies have focused on the role of the FoxA protein Fork head (Fkh) in the embryonic and larval salivary gland (SG), although as with the worm PHA-4 and mammalian FoxA genes, Fkh is expressed in multiple embryonic cell types (Figure 1A). In SGs, Fkh expression is activated by a homeotic protein, Sex combs reduced (Scr), and two other cofactors, Extradenticle (Exd) and Homothorax (Hth) (Figure 1B) [24], [25]. Fkh plays many key roles in the SG, including keeping the SG cells alive through repression of the proapoptotic genes *reaper* and *hid* [26], [27], mediating the cell shape changes of SG invagination required to form the SG tubes [27], and working with the SG-specific bHLH protein Sage to regulate two downstream target genes, *PH4αSG1* and *PH4αSG2*, which are required to maintain uniform patent SG lumens [28] (Figure 1C). Fkh also regulates its own expression and maintains expression of two other SG transcription factors, CrebA and Sage [28], [29], [30]. In vitro binding studies reveal that Fkh binds to the same consensus sites that have been described for the vertebrate FoxA proteins and worm PHA-4, but that certain residue combinations in key flanking positions disrupt binding [31]. Studies of endogenous Fkh binding sites in vitro and regulation of the corresponding target genes in vivo support these findings [28], suggesting that the requirements for in vivo binding and regulation by FoxA proteins may be relatively stringent. Indeed, recent studies reveal that binding of both mammalian FoxA and worm PHA-4 to consensus sites is similarly affected by flanking sequences [32], [33].

Although several key SG targets of *Drosophila* Fkh have been analyzed to date, there have been no genome-wide surveys to identify the range of Fkh targets. Here, we use two approaches to obtain a more global view of *Drosophila* Fkh targets in both the SG and the whole embryo. Our studies demonstrate that Fkh plays a major role in SG maintenance and function. These studies also reveal unexpected roles for Fkh in endoreduplication and in blocking terminal gene expression in early embryos.

## Results

### Fkh plays a major role in maintaining salivary gland fate and function

**Figure 2:**
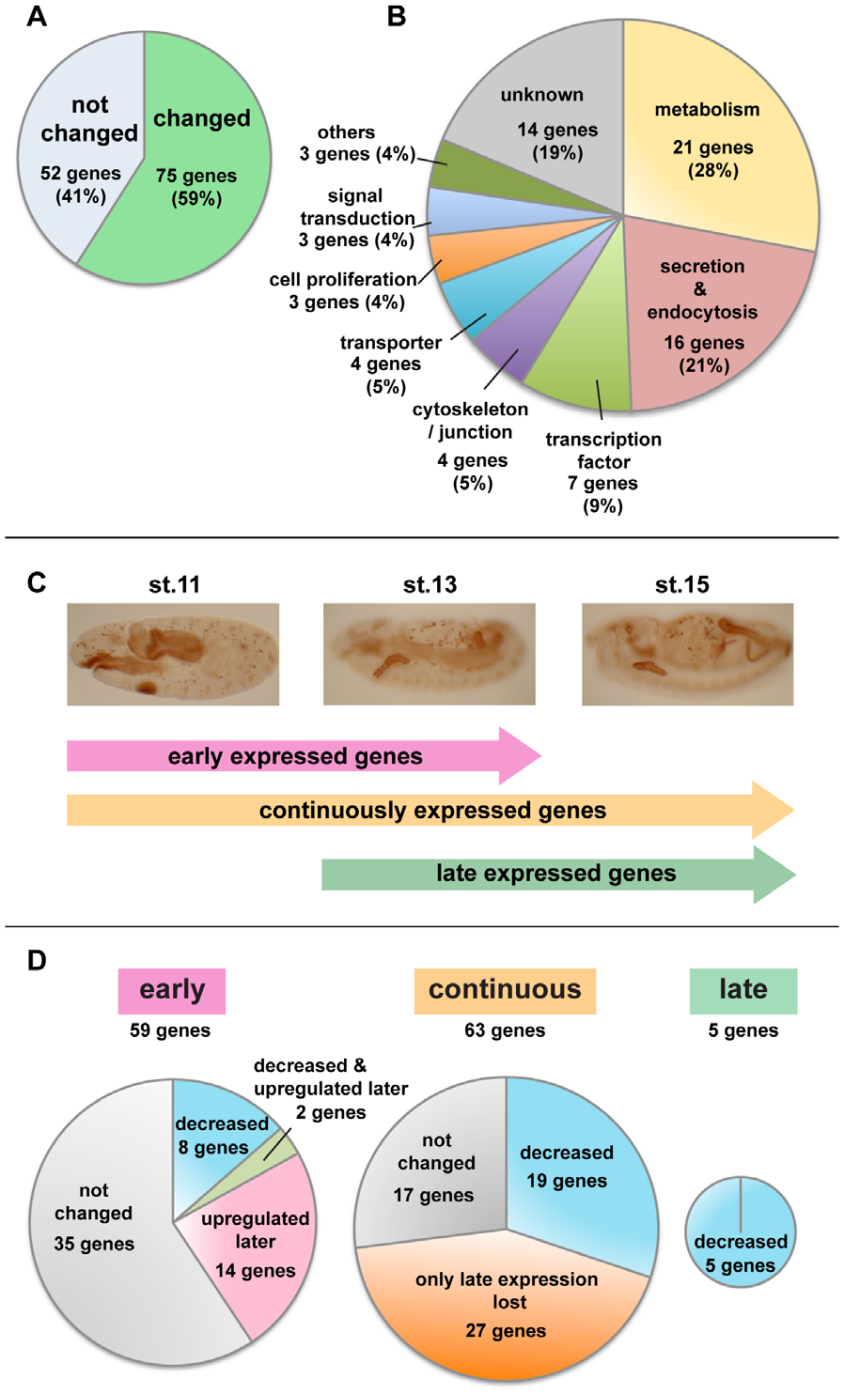
In situ hybridization analysis reveals that Fkh affects expression of many salivary gland genes. (A) Fkh is required for normal expression of 75 out of 127 tested SG genes. (B) Gene ontology (GO) terms associated with the SG Fkh downstream genes reveal that Fkh regulates genes implicated in a variety of activities, including metabolism and secretion/ endocytosis. (C) SG genes can be categorized based on their temporal patterns of expression. (D) Fkh affects the expression of fewer early genes than late genes. Expression of only 40% of early genes is affected by *fkh* loss (left), whereas expression of 71% of continuously expressed genes (middle) and expression of 100% of late expressed genes are affected by *fkh* loss (right). Importantly, most SG genes start to express during stage 10 or 11 (122/127), and the ‘upregulated later’ group and ‘only late expression lost’ group are the largest groups of Fkh dependent genes in each category, indicating that Fkh regulates more SG genes in late stages.

To determine what proportion of salivary gland (SG) genes depend on Fkh for their expression, we performed a large-scale in situ hybridization analysis of SG gene expression in WT and *fkh* loss-of-function mutant embryos. SG genes were chosen from the expression pattern database Release 2 of the Berkeley Drosophila Genome Project (BDGP [http://www.fruitfly.org/cgi-bin/ex/insitu.pl]), which is in the process of determining the embryonic expression patterns of all *Drosophila* genes. Whole mount in situ hybridization analysis of 190 different genes in WT embryos revealed 127 with reliable SG expression. Of the 127 genes, 59% (75 genes) had altered expression patterns in *fkh* mutants (Figure 2A). Approximately half of the affected genes encode products related to SG function based on published data and Gene Ontology assignments: metabolism, secretion and endocytosis (Figure 2B). Other genes affected by loss of *fkh* encode a variety of proteins, suggesting that Fkh plays a major role in both SG development and function.

Analysis of the temporal expression patterns of the SG genes in WT and *fkh* mutants revealed that Fkh affects late gene expression far more than it does early gene expression. SG genes were grouped into three classes based on their WT expression pattern (Figure 2C). “Early expressed genes” (59 genes) included those expressed beginning at stage 10, when Fkh protein is initially detected, and ending at around stage 13. “Continuously expressed genes” (63 genes) were expressed beginning at stage 10 or 11 and continuing through embryogenesis. The “late expressed genes” (5 genes) were expressed beginning after stage 12 and continuing through embryogenesis. 73% (46/63 genes) of continuously expressed genes and 100% (5/5 genes) of late expressed genes were dependent on Fkh for their expression (Figure 2D), whereas only 41% (24/59 genes) of early expressed SG genes were affected by *fkh* loss (Figure 2D).

**Figure 3:**
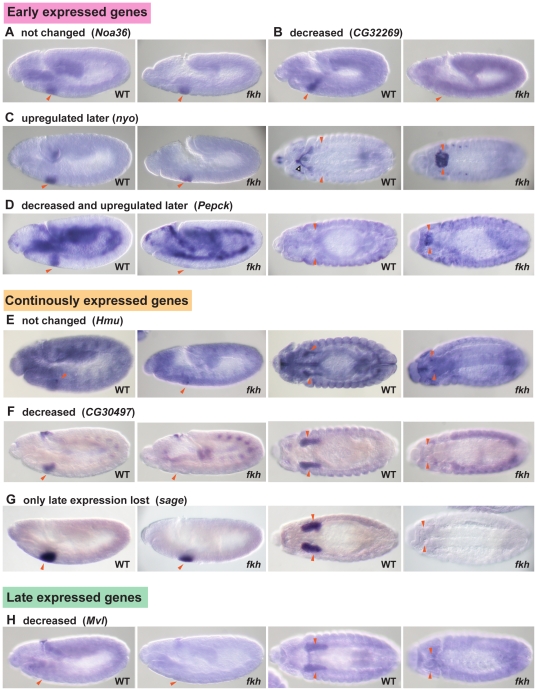
Six groups of *fkh* dependent genes. In situ hybridizations of examples from each group of SG genes is shown for WT and *fkh* mutants. The *fkh* mutants are homozygous for *Df(3L)H99*, which blocks the SG cell death associated with *fkh* loss. Red arrowheads indicate the SGs. Blank arrowheads indicate the salivary duct. (A,B) Lateral views of WT and *fkh* embryos at stage 11 are shown. (C–H) Left two panels show lateral views of WT and *fkh* embryos at stage 11. Right two panels show ventral views of WT and *fkh* embryos at stage 13/14. SGs remain on the ventral surface of *fkh* mutants. (A) Fkh does not affect expression of most early SG genes, including *Noa36*. (B) Expression of eight early SG genes was significantly reduced in SGs of *fkh* mutants, as shown with *CG32269*. (C) Fourteen early genes affected by loss of *fkh* showed higher levels of expression in late embryonic SGs. This group included *trh* and *nyo*, which are initially expressed throughout the SG and duct primordia but that subsequently become restricted to the duct in WT embryos. (D) Two early expressed SG genes showed decreased expression at early stages and increased expression at late stages, as shown for the *Pepck* gene. (E) Expression of 27% of continuously expressed SG genes was unaffected by *fkh* loss. The *Hmu* gene is an example of this class. *Hmu* transcripts localize to the apical domains of SG cells. (F) 30% of continuously expressed SG genes showed reduced expression at all stages in *fkh* mutants, as seen with *CG30497*. (G) Many continuously expressed genes were unaffected at early stages but showed reduced expression at late stages, including the bHLH transcription factor gene *sage*. (H) All five late expressed SG genes were affected by loss of *fkh*, as observed with *Mvl*.

Further analysis revealed additional subtlety in how the early and continuously expressed classes of genes were affected by *fkh* loss. Early expressed genes affected by *fkh* loss could be classified into three groups, ‘decreased’, ‘upregulated later’ and ‘decreased and upregulated later’ (Figure 2D and 3A–D). Continuously expressed Fkh dependent genes could be classified into two groups, ‘decreased’ and ‘only late expression lost’ (Figure 2D and 3E–G). All late expressed genes were similarly affected by loss of *fkh* and showed decreased expression (Figure 2D and 3H). The variety of expression changes in *fkh* mutants suggests that Fkh can both activate and repress gene expression and that SG genes are differentially regulated between early and late stages of development.

The ‘decreased’ group of early expressed genes (eight genes) includes those whose expression was significantly reduced or gone in the SGs of *fkh* mutants (Figure 3B). These genes are good candidates for direct regulation by *fkh*; moreover, their early transient expression also suggests a potential role in SG morphogenesis. The ‘upregulated later’ group of early expressed genes (14 genes) showed no change in expression in SGs between WT and *fkh* mutants at stage 11; however, expression persisted much longer in *fkh* mutants than in WT (Figure 2D and 3C), suggesting repression by Fkh at later stages. Interestingly, four transcription factor-encoding genes are included in this group (Figure 2B). One of these genes was *trachealess* (*trh*), which encodes a bHLH-PAS transcription factor required for the development of the salivary gland ducts [34]. As observed with other known duct genes, *trh* is expressed initially in both the duct and secretory gland primordia until stage 12 when Fkh downregulates its expression in the secretory cells [34], [35]. Another gene in the ‘upregulated later’ group, *nyobe* (*nyo*) [36] showed an expression pattern similar to that of *trh*, although its expression persisted in the proximal secretory cells in WT SGs to stage 13 (Figure 3C). Two possibilities exist for regulation of *nyo* by Fkh: (1) like *trh*, *nyo* could be repressed by Fkh in late secretory cells or (2) *nyo* expression in duct cells could be activated by Trh and only indirectly repressed by Fkh. The remaining genes in the ‘upregulated later’ group are not expressed in the duct or duct primordia, suggesting that Fkh also represses late expression of a subset of early secretory cell specific genes, including other transcription factor genes. Expression of the ‘decreased and upregulated later’ group of early SG genes (two genes) was decreased or diminished at stage 11 but was also detected in SGs of *fkh* mutants at stage 13, when expression of these genes in WT SGs had disappeared (Figure 2D and 3D).

**Figure 4:**
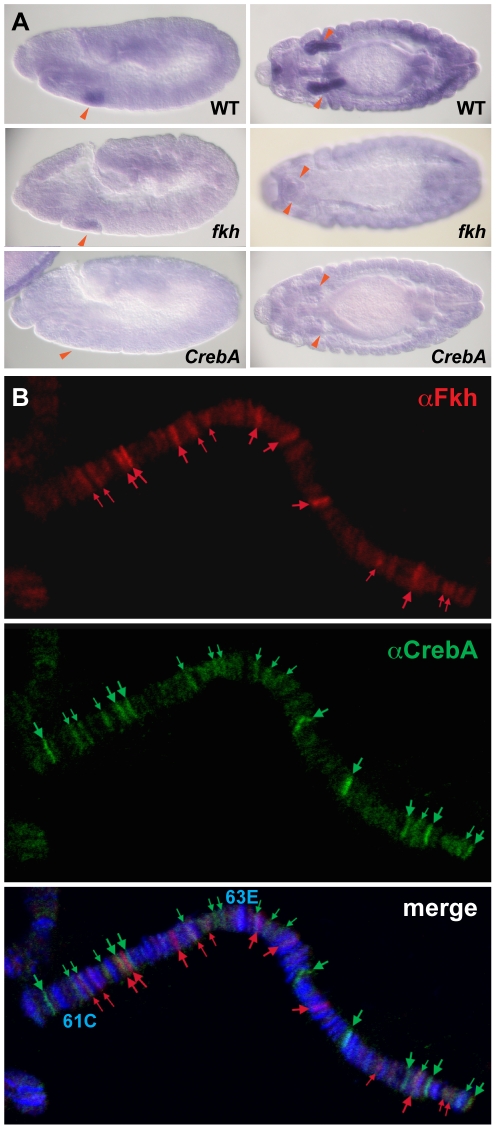
Fkh regulates some SG genes indirectly through maintaining CrebA expression. (A) In situ hybridization of the *baiser* gene in WT, *fkh H99*, *CrebA* mutants. As shown here with *baiser*, many genes whose SG expression disappears only in late *fkh* mutants require CrebA for their SG expression at all times. Red arrowheads: SGs. (B) Fkh and CrebA bind different sites on SG polytene chromosomes, suggesting that regulation of target genes does not involve cooperative regulation by Fkh and CrebA. Red: αFkh, green: αCrebA, blue: DAPI.

Salivary gland expression of the 19 continuously expressed genes in the ‘decreased’ group was either significantly decreased or gone at all stages in *fkh* mutants (Figure 2D and 3F). These genes are also good candidates for direct regulation by Fkh. Among this group of targets is *PH4αSG2*, which encodes an ER enzyme whose expression has been shown to be directly activated by Fkh [28]. The ‘only late expression lost’ group of continuously expressed genes (27 genes) is likely to include both direct and indirect Fkh targets (Figure 2D and 3G). An example of a direct target in this group is *CrebA*, which encodes a bZip transcription factor required for increased secretory capacity [29], [37]. *CrebA* expression is initially activated by the same transcription factors that activate *fkh* expression in the SG - Scr, Exd and Hth. Both CrebA and Fkh subsequently become directly dependent on Fkh for their maintained expression, since expression of Scr, Exd and Hth disappears early as the SG cells begin to invaginate [25], [29], [30]. Examples of likely indirect targets in the ‘only late expression lost’ group are 18 genes whose expression is also downregulated in *CrebA* mutants, based on in situ and/or microarray analysis [29], [37]. Twelve CrebA target genes in the ‘only late expression lost’ group have been categorized as being involved in secretion and endocytosis based on Gene Ontology assignments, including *baiser* (*bai*) (Figure 4A), which encodes a p24 protein family member involved in ER-Golgi transport [38]( Table S1). Given previous findings that Fkh directly maintains CrebA expression in the SG [29] and that CrebA has been shown to directly regulate expression of most secretory genes [37], Fkh may affect expression of these genes only indirectly by maintaining CrebA expression. Importantly, the secretory pathway genes that are also regulated by CrebA represent a large proportion of the ‘only late expression lost’ group.

Since the *C.elegans* Fkh/FoxA homolog PHA-4 is thought to directly activate expression of most or perhaps all pharyngeal-specific genes [23], [39], we asked if CrebA and Fkh might cooperate to directly maintain expression of their target genes in the SG. Since both genes are normally expressed in the larval SG, we simply stained larval SG polytene chromosomes with CrebA and Fkh antibodies and asked if the two proteins bind an overlapping set of sites. As shown in Figure 4B, there is very little overlap in the sites bound by CrebA and Fkh, suggesting that Fkh plays no direct role in the regulation of CrebA SG target genes.

In summary, our large scale in situ analysis reveals that Fkh plays a major role in maintaining SG fate and physiological activity. Fkh affects the majority of SG genes (59%) and loss of *fkh* leads to both decreases and increases in SG gene expression. Our analysis also identified a large number of new Fkh dependent genes, which encode proteins implicated in a wide variety of functions. Furthermore, we found that Fkh regulates the expression of far more genes at late stages than at early stages. This can be explained by a model wherein Fkh regulation of many SG genes is indirect and through its role in maintaining the expression of other SG transcription factors such as CrebA.

### Fkh is not an “organ-specifying” gene

**Figure 5:**
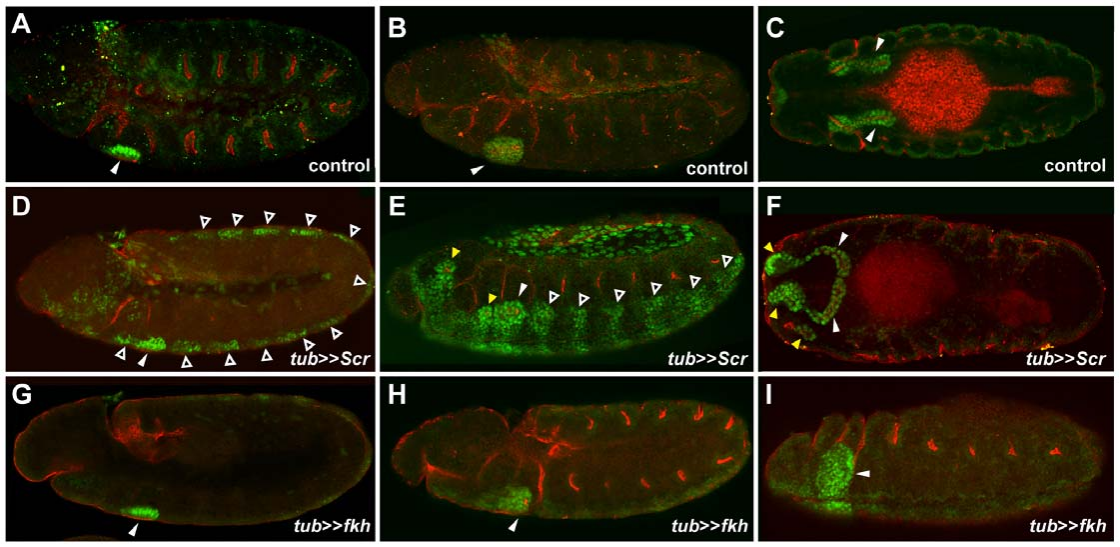
Fkh overexpression does not induce SGs in additional embryonic segments. Embryos were immunostained with αCrebA (Green) and αCrb (red). (A–C) SGs form in only parasegment 2 (PS2) in wild type (not shown), *tubulin(tub)-Gal4*/+ (A–C) or UAS-*fkh*/+ (not shown) control embryos (white arrowheads). (D–F) *tub-Gal4* driven expression of UAS-*Scr* results in transient upregulated expression of CrebA in almost every segment of the embryo (open arrowheads). Only the high-level CrebA expressing cells of parasegments 0 and 1 invaginate and make SGs (yellow arrowheads) in addition to the endogenous glands in parasegment 2 (white arrowhead). (G–H) *tub-Gal4* driven expression of UAS-*fkh* activates very low-level expression of CrebA in almost all segments of the embryo but does not result in the formation of additional glands. High-level expression of Fkh also disrupts invagination of the glands that normally form in PS2 (white arrowhead).

Our large scale in situ analysis revealed that Fkh affects the expression of 59% of SG genes. In contrast, the hox protein Scr, which functions upstream of Fkh, is required for expression of every SG gene that has been tested [24], [40], [41], [42]. Moreover, Scr over-expression using a heat-shock inducible promoter results in the formation of additional SGs in more anterior regions of the embryo (parasegments 0 and 1) [43]. To ask if Fkh can also drive formation of additional SGs, we expressed either Scr or Fkh throughout embryos using UAS-Gal4 system [44]. Consistent with previous findings [43], Scr expressed under the control of the tubulin-Gal4 driver resulted in formation of extra SGs in the head region (Figure 5E, F). Staining of these embryos with antibodies to CrebA and Crb revealed that these extra SGs invaginated and formed epithelial lumens. Transient CrebA expression without Crb signal was also observed in all other embryonic segments at early stages (Figure 5D,E). In contrast, Fkh expression driven by the tubulin-Gal4 driver did not result in the formation of SGs in additional segments (Figure 5G–I), even though CrebA was weakly induced throughout the embryo (Figure 5H). The only additional cells that expressed persistent high level CrebA were the salivary duct primordia, consistent with the previously described role for Fkh in shutting off duct specific gene expression [35]. Unexpectedly, *fkh* overexpression throughout the embryo arrested SG development and disrupted germband retraction (Figure 5I). These data reveal that, unlike Scr, Fkh is insufficient to drive SG formation on its own.

### Microarray studies of whole embryos reveal new roles for Fkh

**Figure 6:**
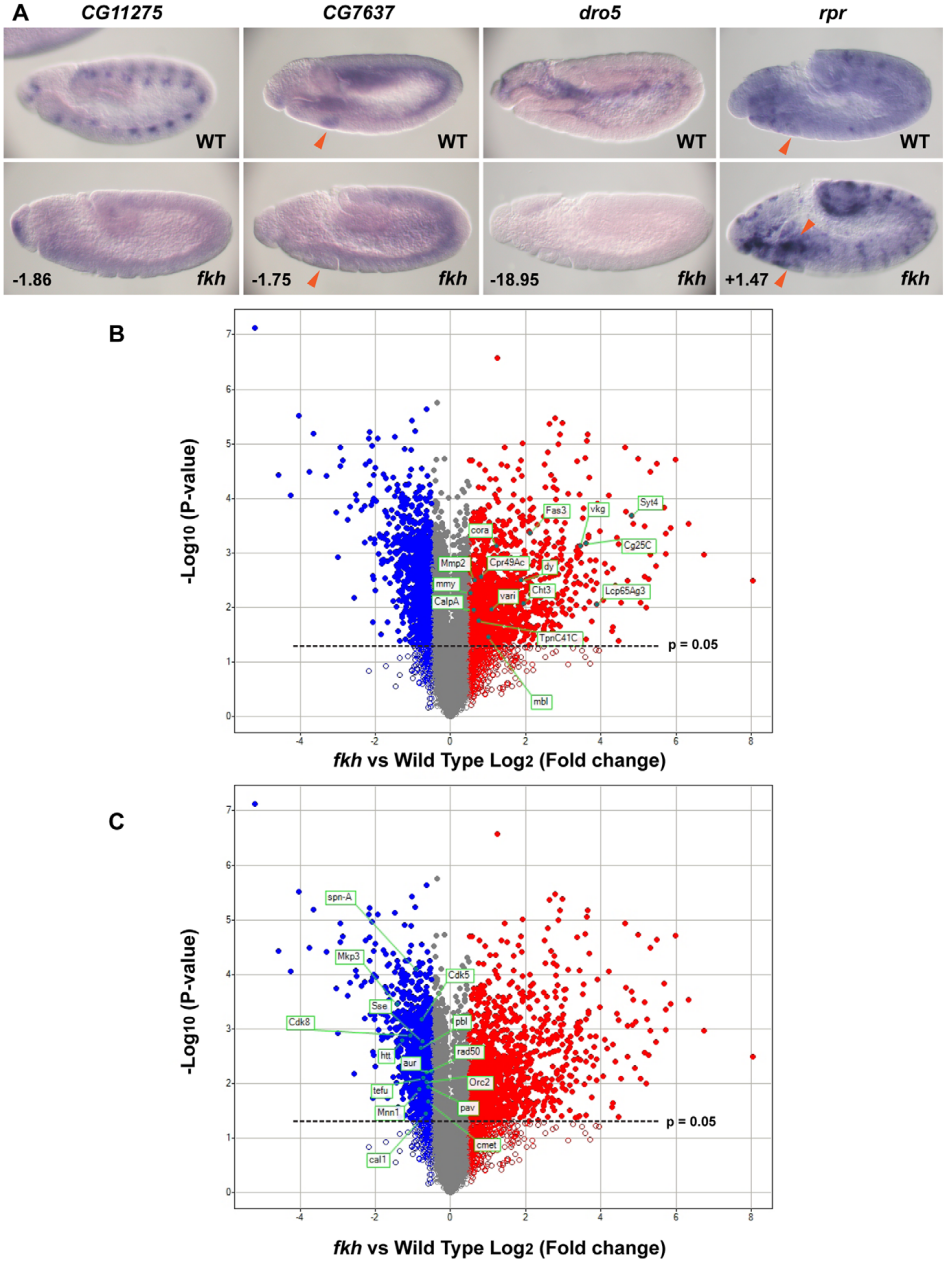
Microarray analysis suggests a role for Fkh in terminal differentiation and endoreplication. (A) Genes identified as both downregulated and upregulated by microarray analysis show the expected expression changes in WT versus *fkh* mutant embryos when examined by whole mount in situ hybridization. *CG11275*, *CG7637* and *dro5* all had notably reduced expression in *fkh* mutant embryos, whereas *rpr* expression was notably higher in *fkh* mutants. The numbers indicate the fold-change of each gene. Red arrowheads: SGs. (B) Volcano plot shows genes whose expression is significantly downregulated (blue filled circles) and upregulated (red filled circles) in *fkh* mutant embryos. Genes whose expression changed but with P-values greater than 0.05 are shown with open circles. Highlighted in green are examples of upregulated genes in *fkh* mutants. Chitin related: *Cht3*; cell junction and synapse: *Syt4*, *Fas3*, *cora*, and *vari*; cuticle: *dy*, *Lcp65Ag3* and *Cpr49Ac*, ECM: *Cg25C*, *vkg*, *mmy*, and *Mmp2*; muscle: *mbl*, Calcium: *CalpA* and *TpnC41C*. (C) The same volcano plot is shown in B with downregulated genes involved in chromosome metabolism and cell cycle progression highlighted in green.

Our in situ hybridization analysis identified a large number of Fkh-dependent SG genes; this analysis will have missed many Fkh targets, however, since the BDGP expression datasets are incomplete (only about 1/3 of the genome is represented so far) and because the in situ hybridization analysis cannot identify genes that might normally be repressed in the early SG by Fkh. Thus, to identify additional *fkh* downstream target genes, we performed a microarray analysis comparing the expression profiles of WT and *fkh* stage 11 embryos. From the microarrays, we discovered 1102 down-regulated genes (with a fold change <−1.4, P<0.05) and 1087 up-regulated genes (with a fold change >1.4, P<0.05) in *fkh* mutants compared to WT (Table S2). To validate the microarray data, we selected a set of both down-regulated and up-regulated genes from the microarray data and performed in situ hybridization analysis of these genes in WT and *fkh* mutant embryos. Five of the seven down-regulated genes we tested had notably reduced expression and five of the nine upregulated genes we tested had notably higher expression in *fkh* mutant embryos (examples are shown in Figure 6A). These findings indicate that the microarray approach can identify new Fkh dependent genes in the SG and many additional cell types in which this transcription factor is expressed.

To ask if the microarray approach was sensitive enough to identify the SG genes we had previously shown to be regulated by Fkh during stage 11, we asked if the Fkh dependent genes identified by in situ analysis were included in the downregulated set identified by microarray. Most of the 28 SG genes affected by *fkh* loss at this stage did not show significant expression changes in the microarray analysis; seven were downregulated, one was upregulated and 20 were unchanged (Figure S1). This finding suggests that genes that are expressed more broadly and but may be regulated by Fkh in only the SG will be missed by this analysis, which averages gene expression changes over the entire embryo (see, for example, Fig. 3D; Figure S1).

To learn more about the types of genes regulated by Fkh in the entire embryo, we analyzed the microarray data using DAVID [45], [46], a program that identifies functional groups (based on Gene Ontology terms) that are enriched in a given dataset compared with their representation in the entire genome. The DAVID analysis revealed that genes involved in terminal differentiation were significantly enriched in the upregulated data set. Examples of these functional groups include chitin and polysaccharide related genes (enrichment score: 6.74), cell junction and synapse genes (enrichment score: 5.52), cuticle genes (enrichment score: 4.90), extracellular matrix genes (enrichment score: 4.04), and muscle genes (enrichment score: 3.48) (Figure 6B, Table 1). These data suggest a general role for Fkh in repressing expression of genes required for terminal differentiation in early embryos.

**Table 1:** Clustering analysis of GO terms for genes upregulated in *fkh* mutants.

Annotation Cluster^a^	Gene Onotology (GO) term	Fold enrichment	P-value
1 (6.74)	Chitin metabolic process	3.6	9.0×10^−9^
	Polysaccharide metabolic process	3.4	1.5×10^−10^
	Aminoglycan metabolic process	3.4	1.4×10^−9^
	Polysaccharide binding	3.0	9.2×10^−8^
	Pattern binding	3.0	9.2×10^−8^
	Chitin binding	3.0	2.7×10^−6^
	Chitin binding protein, peritrophin-A	2.9	1.5×10^−5^
	ChtBD2	2.6	6.8×10^−5^
	Carbohydrate binding	2.4	2.4×10^−6^
	Extracellular region	1.8	3.2×10^−7^
2 (5.52)	Synapse	3.8	8.3×10^−6^
	Cell junction	3.2	1.5×10^−5^
	Cell junction	3.0	9.8×10^−8^
	Synapse	2.9	1.0×10^−6^
	Synapse part	2.8	1.9×10^−5^
3 (4.90)	Structural constituent of cuticle	3.0	1.6×10^−7^
	Structural constituent of chitin-based larval cuticle	2.9	1.1×10^−2^
	Insect cuticle protein	2.8	4.4×10^−6^
	Structural constituent of chitin-based cuticle	2.8	4.4×10^−6^
	Structural molecule activity	1.8	9.1×10^−6^
4 (4.04)	Basement membrane	7.8	1.0×10^−4^
	Extracellular matrix part	7.2	1.8×10^−4^
	Extracellular matrix	4.0	4.2×10^−6^
	Proteinaceous extracellular matrix	4.0	1.0×10^−5^
	Extracellular region part	1.9	7.9×10^−3^
5 (3.98)	Fatty acid biosynthetic process	5.1	2.7×10^−5^
	Lipid synthesis	4.4	8.9×10^−3^
	Fatty acid metabolic process	4.1	9.2×10^−6^
	Organic acid biosynthetic process	3.6	2.1×10^−5^
	Carboxylic acid biosynthetic process	3.6	2.1×10^−5^
	Lipid biosynthetic process	2.3	1.3×10^−3^
6 (3.60)	Plasma membrane part	2.0	3.1×10^−8^
	Integral to plasma membrane	1.6	2.1×10^−2^
	Intrinsic to plasma membrane	1.6	2.5×10^−2^
7 (3.48)	Muscle	13	1.8×10^−5^
	Striated muscle thin filament	13	1.4×10^−4^
	Contractile fiber	10	3.3×10^−12^
	Sarcomere	9.8	1.0×10^−8^
	Contractile fiber part	9.7	3.1×10^−10^
	Muscle protein	9.4	2.4×10^−9^
	Myofibril	9.2	2.5×10^−8^
	Myofibril assembly	7.2	2.0×10^−4^
	Skeletal muscle tissue development	3.7	2.2×10^−3^
	Skeletal muscle organ development	3.3	4.2×10^−4^
	Striated muscle tissue development	3.3	5.3×10^−3^
	Striated muscle cell development	3.2	3.1×10^−3^
	Muscle cell development	3.2	3.1×10^−3^
	Muscle tissue development	3.2	6.2×10^−3^
	Striated muscle cell differentiation	3.1	4.1×10^−4^
	Muscle cell differentiation	2.9	4.6×10^−4^
	Actomyosin structure organization	2.7	4.4×10^−2^
	Actin cytoskeleton	2.3	4.6×10^−3^
	Muscle organ development	2.1	3.4×10^−3^
8 (3.00)	Domain: EF-hand 3	6.6	2.0×10^−5^
	Domain: EF-hand 4	6.1	1.8×10^−3^
	Domain: EF-hand 1	4.9	9.0×10^−5^
	Domain: EF-hand 2	4.9	9.0×10^−5^
	Calcium-binding region 1	4.8	7.8×10^−4^
	Calcium-binding region 2	4.8	7.8×10^−4^
	EF hand	4.8	1.6×10^−2^
	Signal transduction mechanisms / Cytoskeleton / Cell division and chromosome partitioning / General function prediction only	3.3	1.5×10^−2^
	Calcium	2.4	1.9×10^−5^
	Calcium ion binding	2.3	4.1×10^−7^
	EF-Hand 1	2.1	3.2×10^−3^
	EF-Hand type	2.1	3.7×10^−3^
	Calcium-binding EF-hand	2.1	2.2×10^−2^
	EF hand	2.1	2.2×10^−2^
	EF-Hand 2	2.0	1.1×10^−2^
	EFh	1.8	4.5×10^−2^

David analysis reveals a significant enrichment of eight annotation clusters (based on Gene Ontology terms) in the set of genes upregulated in stage 11 *fkh* mutant embryos. Shown are all clusters with enrichment scores ≥3.00

a The enrichment score is shown in parentheses. P<0.05.

DAVID analysis of the genes that were downregulated in *fkh* mutants revealed a significant enrichment of chromosome and cell cycle related genes (Figure 6C, Table 2). Examples of enriched functional groups include DNA metabolic process (enrichment score: 20.20), chromosome (enrichment score: 7.28) and cell cycle (enrichment score: 3.30). These findings suggest an unexpected role for Fkh in cell cycle progression. Only two of the top ten enriched annotation clusters in the genes downregulated in *fkh* mutants were not related to chromosomes and cell cycle. One enriched cluster was linked to transcription (enrichment score: 3.44) and one was linked to lifespan (enrichment score 3.40).

**Table 2:** Clustering analysis of GO terms for genes downregulated in *fkh* mutants.

Annotation Cluster^a^	Gene Onotology (GO) term	Fold enrichment	P-value
1 (20.20)	DNA repair	5.2	2.7×10^−20^
	Response to DNA damage stimulus	5.0	1.7×10^−21^
	DNA metabolic process	4.1	2.2×10^−27^
	Cellular response to stress	3.4	1.6×10^−14^
2 (7.28)	Chromosome	2.4	4.7×10^−11^
	Chromosomal part	2.4	6.3×10^−9^
	Intracellular non-membrane-bounded organelle	1.5	5.2×10^−6^
	Non-membrane-bounded organelle	1.5	5.2×10^−^^6^
3 (5.23)	Nuclear chromosome	3.4	1.7×10^−^^6^
	Nuclear chromosome part	3.4	5.2×10^−^^6^
	Nuclear chromatin	2.7	2.3×10^−^^2^
	Chromosomal part	2.4	6.3×10^−^^9^
4 (4.34)	DNA recombination	5.6	3.3×10^−^^9^
	Reciprocal meiotic recombination	3.6	1.0×10^−^^2^
	Meiosis I	3.0	2.7×10^−^^3^
5 (3.77)	Nucleoside binding	1.5	9.1×10^−^^6^
	ATP binding	1.5	9.7×10^−^^6^
	Adenyl ribonucleotide binding	1.5	1.1×10^−^^5^
	Adenyl nucleotide binding	1.5	1.3×10^−^^5^
	Purine nucleoside binding	1.5	1.7×10^−^^5^
	ATP-binding	1.5	3.4×10^−^^4^
	Purine nucleotide binding	1.4	2.1×10^−^^4^
	Ribonucleotide binding	1.4	2.6×10^−^^4^
	Purine ribonucleotide binding	1.4	2.6×10^−^^4^
	Nucleotide binding	1.3	5.0×10^−^^4^
	Nucleotide-binding	1.3	1.9×10^−^^2^
6 (3.48)	Double-strand break repair via homologous recombination	8.8	1.2×10^−^^3^
	Recombinational repair	8.8	1.2×10^−^^3^
	Double-strand break repair	7.1	1.5×10^−^^7^
7 (3.44)	DNA binding	1.8	7.5×10^−^^11^
	DNA binding	1.8	7.8×10^−^^6^
	Regulation of transcription from RNA polymerase II promoter	1.7	1.2×10^−^^2^
	Nucleus	1.6	6.1×10^−^^7^
	Transcription	1.6	9.0×10^−4^
	Transcription regulation	1.6	1.1×10^−3^
	Transcription	1.5	1.8×10^−3^
	Regulation of transcription	1.4	2.2×10^−4^
	Transcription regulator activity	1.3	1.4×10^−2^
	Regulation of RNA metabolic process	1.3	3.4×10^−2^
	Regulation of transcription, DNA-dependent	1.3	4.7×10^−2^
8 (3.40)	Aging	2.5	3.9×10^−4^
	Determination of adult life span	2.5	3.9×10^−4^
	Multicellular organismal aging	2.5	3.9×10^−4^
9 (3.30)	Female meiosis chromosome segregation	4.4	4.5×10^−5^
	Meiotic chromosome segregation	3.5	3.1×10^−5^
	Female meiosis	3.3	2.8×10^−5^
	Meiosis I	3.0	2.7×10^−3^
	Chromosome segregation	2.7	5.1×10^−6^
	Spindle organization	1.8	2.2×10^−3^
	M phase of meiotic cell cycle	1.8	2.8×10^−3^
	Meiosis	1.8	2.8×10^−3^
	Meiotic cell cycle	1.8	3.2×10^−3^
	Microtubule cytoskeleton organization	1.7	9.3×10^−4^
	Mitotic spindle organization	1.7	1.3×10^−2^
	Cell cycle	1.6	5.4×10^−5^
	Cell cycle process	1.6	5.7×10^−5^
	Cell cycle phase	1.6	3.0×10^−4^
	Microtubule-based process	1.6	1.6×10^−3^
	Mitotic cell cycle	1.6	1.8×10^−3^
	M phase	1.5	5.9×10^−4^
	Cytoskeleton organization	1.4	6.1×10^−3^
10 (3.21)	Non-recombinational repair	9.9	4.7×10^−3^
	Double-strand break repair via nonhomologous end joining	9.3	3.5×10^−2^
	Telomere capping	8.2	8.7×10^−3^
	Non-homologous end-joining	7.5	1.1×10^−2^
	Telomere maintenance	7.4	6.8×10^−6^
	Telomere organization	7.4	6.8×10^−6^
	Anatomical structure homeostasis	4.0	2.1×10^−5^
	Homeostatic process	2.0	1.3×10^−3^

David analysis reveals a significant enrichment of ten annotation clusters (based on Gene Ontology terms) in the set of genes downregulated in stage 11 *fkh* mutant embryos. Shown are all clusters with enrichment scores ≥3.00

a The enrichment score is shown in parentheses. P<0.05.

### Fkh is required for polytenization of embryonic tissues

**Figure 7:**
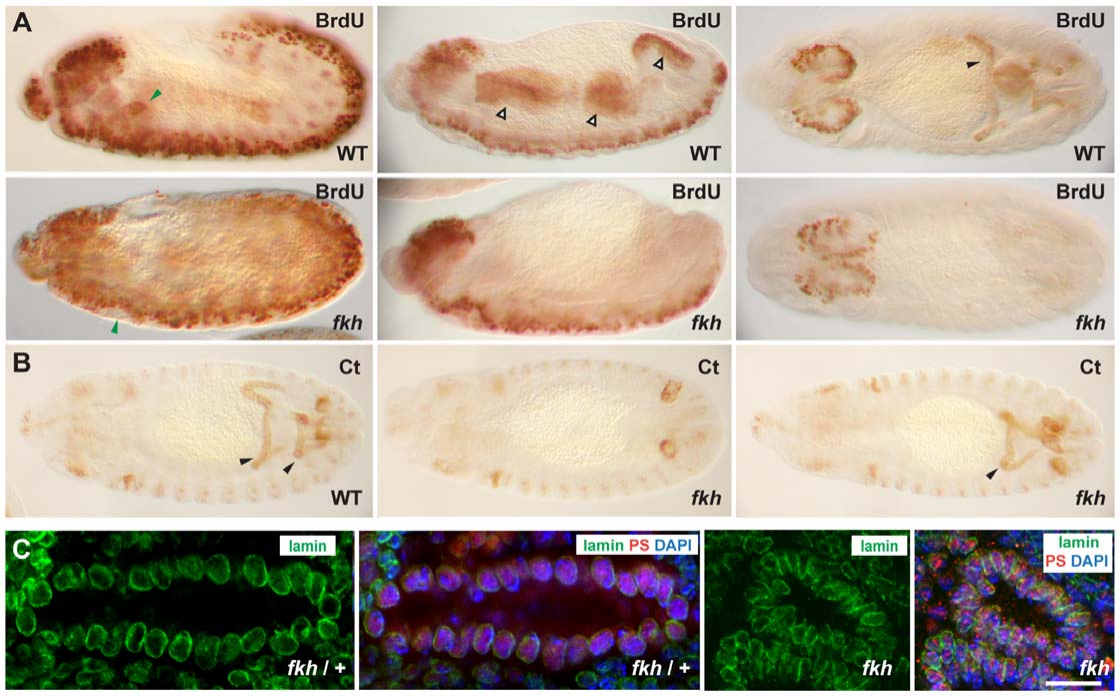
Fkh is required for polytenization in the embryos. (A) The CNS cells of both WT and *fkh H99* mutant embryos undergo normal mitotic cycles throughout embryogenesis and encorporate BrdU. BrdU encorporation is also observed in cells that undergo endocycles in WT embryos (The filled green arrowheads indicate SGs; open arrowheads indicate anterior and posterior midgut and the hindgut; the filled black arrowhead indicates Malpighian tubules). BrdU encorporation is not observed in these tissues in *fkh H99* mutant embryos. (B) Staining of WT and *fkh H99* mutant embryos with the Cut (Ct) antibody, which labels the Malpighian tubules (MTs), reveals that these cells are present in about half of the *fkh H99* mutant embryos, although *fkh H99* mutants showed variable MT defects. Elongated MTs are visible in WT embryos stained with Ct antibodies (left panel). MT staining is absent in some *fkh H99* mutant embryos (middle panel). Other *fkh* mutants show variable defects in MT elongation (right panel). (C) Staining of *fkh H99* heterozygous SGs (two left panels) and *fkh H99* homozygous mutant SGs (two right panels) with nuclear αLamin, αPasilla (SG marker) and DAPI reveals that *fkh H99* heterozygous SG nuclei are larger than *fkh* mutant SG nuclei, consistent with a failure of the *fkh* mutant SGs to undergo normal endocycling. Bar: 10 µm.

The microarray data suggested a potential role for Fkh in activating genes required for cell cycle progression. Interestingly, most larval tissues cease normal mitotic divisions relatively early in embryogenesis; larval cells grow by increases in cell size rather than cell number. Indeed, by embryonic stage 11, only the larval neuroblasts continue to proliferate. All other larval tissues, including the SG, undergo the process of endoreduplication, wherein the DNA replicates but the DNA strands do not separate. This process leads to the formation of polytene chromosomes, a well-known feature of the larval SGs. The large polytenized chromosomes provide templates for the synthesis of RNAs and corresponding proteins that are required in the larger larval cells. The patterns of endoreduplication in embryos, which have been determined by assaying BrdU encorporation [47], correspond quite well with the pattern of Fkh expression; endoreduplication occurs in the SG, anterior and posterior midgut (AMG and PMG), hindgut (HG) and Malpighian tubules (MT), embryonic tissues that express high levels of Fkh (Figure 1A). To ask if Fkh is required for endoreduplication, we carried out BrdU labeling of WT and *fkh* embryos, which marked not only the proliferating CNS cells but also the endoreplicating cells of the SGs, midgut, hindgut, and MTs in WT embryos (Figure 7A). In contrast, *fkh* embryos showed BrdU encorporation only in the CNS revealing that *fkh* is required for endoreplication (Figure 7A). To rule out indirect effects through changes in cell fate, we stained WT and *fkh* mutant embryos with the MT differentiation marker Cut [48]. The MTs of *fkh* mutants stained with Cut about half of the time (Figure 7B) indicating that the failure of MTs to encorporate BrdU, which occurs in 100% of *fkh* mutants, is not due to a change in MT cell fate. Interestingly, the Cut staining also revealed abnormal MT morphologies in *fkh* mutants, indicating a new role for this FoxA protein (Figure 7B).

Since DNA content correlates with nuclear size, we also stained embryos with a nuclear lamin antibody to outline the nuclear membrane and with Pasilla antiserum [41] to mark SG nuclei, to ask if loss of *fkh* affected SG nuclear size. *fkh* mutant SG nuclei were consistently smaller than those of their heterozygous (wild-type) siblings (Figure 7C), in agreement with a failure of *fkh* mutant SGs to undergo endoreduplication. Thus, the microarray data have revealed a new role for *fkh* in endoreduplication, the process required to create the polytenized chromosomes of larval tissues.

## Discussion

In this work, we used genome-wide approaches to identify the transcriptional targets of the *Drosophila* FoxA protein Fkh in the SG and entire embryo. The in situ analysis revealed that Fkh plays a major role in maintaining SG cell fate and affects expression of the majority of SG genes (∼59%). Through the detailed analysis of expression changes, we learn that Fkh both upregulates and downregulates SG gene expression and that regulation of many SG target genes is indirect. We also show that Fkh is not sufficient to drive SG development on its own, a finding consistent with a role in maintaining but not specifying the SG fate. Our whole embryo microarray experiments comparing transcripts from early WT and *fkh* mutant embryos revealed two unexpected findings: (1) that Fkh represses inappropriate expression of terminal differentiation genes in early embryos and (2) that Fkh is required for endoreduplication in the SGs and in other Fkh expressing tissues.

### Role of the FoxA proteins PHA-4 and Fkh in organogenesis

The findings from our in situ analysis of *fkh* versus wild type SGs suggest an alternative paradigm for how FoxA proteins regulate organ-specific gene expression. There are profound differences in how the fly FoxA protein Fkh regulates SG gene expression and how the worm PHA-4 regulates pharyngeal gene expression; however, there are some similarities. Both PHA-4 and Fkh regulate many tissue-specific genes; PHA-4 regulates 100% of pharyngeal genes and Fkh affects expression of the majority of SG genes (59%). Both PHA-4 and Fkh regulate expression of other transcription factors that contribute to tissue formation and/or physiological activity. For example, both proteins activate expression of bHLH proteins that work with them to activate expression of tissue-specific downstream target genes; HLH-6 in the case of PHA-4 [49], [50] and Sage in the case of Fkh [28]. Finally, both FoxA proteins also repress expression of transcription factors that are linked to alternative cell fates; PHA-4 represses expression of the ectodermal regulator LIN-26 and Fkh represses expression of Trachealess (Trh), a key factor in salivary duct formation [34], [51].

Our findings also reveal that PHA-4 and Fkh play different roles in the two organs. Fkh plays a major role in maintaining and implementing the SG cell fate. This role is critically important in this organ since the factors that initiate the SG cell fate – Scr, Exd and Hth – disappear shortly after the glands begin to form [25]. Fkh maintains cell fate, in large part, by maintaining its own expression as well as the expression of at least two other SG-specific transcription factors – CrebA and Sage. Fkh implements the SG cell fate decision by regulating genes required for morphogenesis and by collaborating with other tissue specific factors, such as Sage, to activate SG specific enzymes and gene products [28] (A. Vaishnavi et al., in prep.). Fkh is neither necessary nor sufficient to specify the SG, whereas loss-of-function and overexpression experiments suggest that PHA-4 is both necessary and sufficient to specify, maintain and implement pharyngeal cell fates [20], [21], [51]. Thus, it is not surprising that 100% of pharyngeal genes will be affected by loss of *pha-4*; similar changes in SG gene expression are observed with the loss of *Scr*, the hox gene that specifies the SG.

Finally and importantly, many of the genes affected at late stages are likely to be only indirectly regulated by Fkh. This is certainly the case for most, if not all, of the SG transcriptional targets of the CrebA transcription factor, which mediates high-level secretory capacity by upregulating genes encoding components of secretory organelles [29], [37]. Evidence for indirect regulation of these genes by Fkh through CrebA includes the following: (1) Fkh is required only for late expression of secretory genes, whereas CrebA is required at all stages [29]. (2) Fkh is required to maintain but not initiate CrebA expression in the SG [29]. (3) In vitro binding studies and in vivo expression studies reveal that CrebA binding sites are required for expression of secretory pathway genes [37]. (4) In the larval SG, endogenous Fkh and CrebA bind to largely non-overlapping sites, suggesting that binding of these factors is not interdependent and that they do not work together to activate target genes (Figure 4B). (5) Ectopic expression of CrebA alone is sufficient to induce high-level secretory gene expression in multiple other cell types, including in the trachea, salivary duct, midline glia and ectodermal stripes, tissues that do not express Fkh [37].

Discovering profound differences in both the role and mode of action of FoxA proteins in these two simple models provides alternative paradigms for considering how the mammalian proteins function in the many cell types in which they are expressed and required. Already, studies suggest that mammalian FoxA proteins also function at multiple levels at various tissues. During midbrain dopaminergic (mDA) neuron development, Foxa1 and Foxa2 activate the bHLH transcription factor Neurogenin 2, which is required for cell fate specification [3]. The FoxA proteins are also required for mDA differentiation and expression of a tyrosine hydroxylase essential for dopamine production [3]. Similarly, FoxA proteins activate early regulators of embryonic pancreatic development, and function in mature β-cells to maintain glucose homeostasis through regulation of insulin secretion [12], [52].

### Roles of Fkh in entire embryos

Although a comparison between the two large-scale approaches to discovering target genes revealed that the in situ hybridization analysis is a better way to find tissue-specific Fkh targets (for example Fkh-dependent SG genes; Figure S1), the microarray analysis uncovered some unexpected roles for Fkh. This whole-genome, whole-embryo approach revealed that Fkh represses expression of genes associated with terminal differentiation at early embryonic stages and activates expression of genes associated with cell cycle progression. Our studies following up on Fkh activation of cell cycle genes revealed a new role for Fkh in endoreduplication, the modified cell cycles that lead to formation of the giant polytene chromosomes, the most well known of which are those from the larval SG.

To our knowledge, Fkh is the first transcription factor to be linked to endoreduplication in fly embryos. Indeed, the only other factor known to affect this process in larval tissues is Sunspot (Ssp), a zinc finger DNA binding protein that is negatively regulated by Wingless signaling and that promotes endoreduplication in larval salivary glands through activation of E2F-1 and PCNA expression [53]. Based on our microarray data, expression of *Ssp*, *E2F-1* and *PCNA* are unaffected in *fkh* mutant embryos, suggesting that Fkh affects this process through independent pathways (Table S2). The Notch signaling pathway is required for endoreduplication in the follicle cells of the ovary [54], [55]. Interestingly, Notch is transiently upregulated in SGs in stage 11 embryos [56], just prior to the first round of SG endoreduplication, and our microarray data reveals that one of the Notch ligands, Delta, is downregulated in *fkh* mutants (Table S2). Thus, Fkh may work through Notch signaling or in parallel to Notch to control endocycles. Since Fkh is persistently expressed in this tissue, we favor a model in which Fkh endows cells with the ability to undergo endocycles and other signaling events determine when those cycles will occur. Perhaps Wg or Notch signaling control the timing in all cells undergoing endocycles. It will be exciting to learn if Fkh’s role in endoreduplication is conserved in other systems that undergo endoreduplication, including, for example, cancer cells.

## Materials and Methods

### Fly strains

The fly strains used in this study include Oregon R, *fkh^6^*, *Df(3L)H99*, *CrebA^wR23^*, *tub-Gal4*, *UAS-Scr* (R.Mann) and *UAS-fkh*. The *UAS-fkh* construct was generated by PCR amplification of the *fkh* ORF from a cDNA clone using primers that introduced an XhoI site at the 5’ end and an XbaI site at the 3’ end of the amplified product. The amplified product was subsequently cloned into XhoI/XbaI cut pUAST [44]. The UAS-*fkh* construct was introduced into the genome by P-element mediated insertion, with injection services provided by Rainbow Transgenics.

### Large scale in situ hybridization analysis

Salivary gland genes for in situ analysis were chosen from the expression pattern database Release 2 of the Berkeley Drosophila Genome Project (BDGP). The corresponding cDNA clones were obtained from collections maintained in the laboratories of Allan Spradling or Phil Beachy or were purchased from the Drosophila Genome Resource Center (DGCR). Digoxygenin-labelled antisense RNA probes were generated and hybridizations were carried out as described [57]. *Df(3L)H99 fkh^6^* homozygous embryos were distinguished from their heterozygous siblings either morphologically or by the absence of *lacZ* hybridization, which is driven from a *Ubx-lacZ* insert on the TM6B balancer chromosome. For some of the early expressed genes, *fkh^6^* homozygous embryos were isolated prior to hybridization using a COPAS Select embryo sorter (Union Biometrica). Homozygous embryos were sorted based on the lack of GFP signal driven by the Twi-GFP on TM3 balancer chromosome.

### Immunohistochemistry

Embryo fixation and staining were performed as described [58]. The primary antibodies used were rabbit αFkh (1∶1000, a gift from S. Beckendorf), rat αCrebA (1∶500)[59], mouse αCrb (1∶10, Cq4, DSHB), mouse αβ-gal (1∶10000, Promega), mouse αCut (1∶50, 2B10, DSHB), rat αPS (1∶500)[41], rat αBrdU (1∶50, Serotec) and mouse αLamin (1∶100, ADL84.12, DSHB). The fluorescent-secondary antibodies (Invitrogen) were used at a dilution of 1∶500. Confocal images were obtained using an LSM510 Meta confocal microscope (Carl Zeiss).

### Polytene chromosome staining

Late third instar larval salivary gland polytene chromosomes were prepared as described [60], except that the second fixation was 50% glacial acetic acid. Rabbit αFkh (a gift from S. Beckendorf) and rat αCrebA [59] were each used at a dilution of 1∶100 and the secondary fluorescent antibodies (Molecular Probes; Carlsbad, CA) were used at a dilution of 1∶200. Controls included staining WT SG chromosomes with preimmune serum and/or staining with no primary serum (when pre-immune serum was unavailable).

### Microarray experiments to compare WT and *fkh* mutants

Total RNA was isolated from three independent collections of stage 11 WT and three independent collections of stage 11 *fkh^6^* mutant embryos sorted by a COPAS Select embryo sorter (Union Biometrica) as previously described [37], labeled, and hybridized to the *Drosophila* genome 2.0 chip (Affymetrix). Following scanning, intensity values were normalized (Partek Inc: Irizarry et al., 2003a, 2003b). Fkh dependent genes were identified based on a 1.4-fold change in gene expression, with p-value <0.05. This fold change was selected because the change in gene expression is being averaged over the entire embryo, which includes a majority of cells that do not express Fkh. All of our microarray data are MIAME compliant and have been deposited in the GEOarchive, accession number GSE28324.

### BrdU labeling

WT and *Df(3L)H99 fkh^6^* embryos were labeled with BrdU following a modification of the protocol as described [47], [61]. For BrdU uptake, embryos were permeabilized by octane for 5 min, and incubated in 1 mg/ml BrdU (Sigma-Aldrich) in Schneider’s media (GIBCO) for 30 min at room temperature. Embryos were subsequently fixed in a 1∶1 mixture of 3.7% formaldehyde in PBS and heptane for 30 min and devitellinized using methanol. For optimal HRP detection, the labeled embryos were incubated in 2M HCl with 0.1% Tween 20 for 40 min and washed in 0.1M Na Borate to stop the reaction. Immunohistochemistry to detect BrdU was carried out as described above.

## Supporting Information

Figure S1 **Volcano plot of gene expression changes between stage 11 WT and** ***fkh*** **mutant embryos.** Genes whose expression is significantly downregulated in *fkh* mutants are shown with blue filled circles and genes whose expression is sifnificantly upregulated in *fkh* mutants are shown with red filled circles. Genes whose expression changed but with P-values greater than 0.05 are shown with open circles. Highlighted in green are Fkh-dependent SG genes identified by our in situ hybridization analysis.
(TIF)

Table S1 **Summary of the results from the large-scale in situ hybridization analysis of SG genes in** ***fkh*** **mutants.** Column 1 indicates the CG assignment, column 2, the gene name if one has been given, column 3, the cDNA used for the in situ analysis, column 4, if expression changed or not, column 5, how expression changed, if it did, column 5, gene function based on Gene Ontology term assignments, and column 6, whether the gene is known to be a CrebA target or not, based on the microarray analysis [37].
(XLS)

Table S2 **Fkh-dependent genes identified by the microarray analysis.** Column 1 indicates the gene symbol or CG assignment, column 2, the gene name or CG assignment, column 3, fold-change in expression (*fkh* vs WT), and column 4, p-value (*fkh* vs WT) based on three independent samples of WT and three independent samples of *fkh* mutant embryos.
(XLS)
