# Supplementary figures and images for: Genome-Wide Analysis Reveals a Major Role in Cell Fate Maintenance and an Unexpected Role in Endoreduplication for the Drosophila FoxA Gene Fork Head
Source: PLoS One. 2011 Jun 16;6(6):e20901. doi: 10.1371/journal.pone.0020901 (PMC3116861; doi:10.1371/journal.pone.0020901)

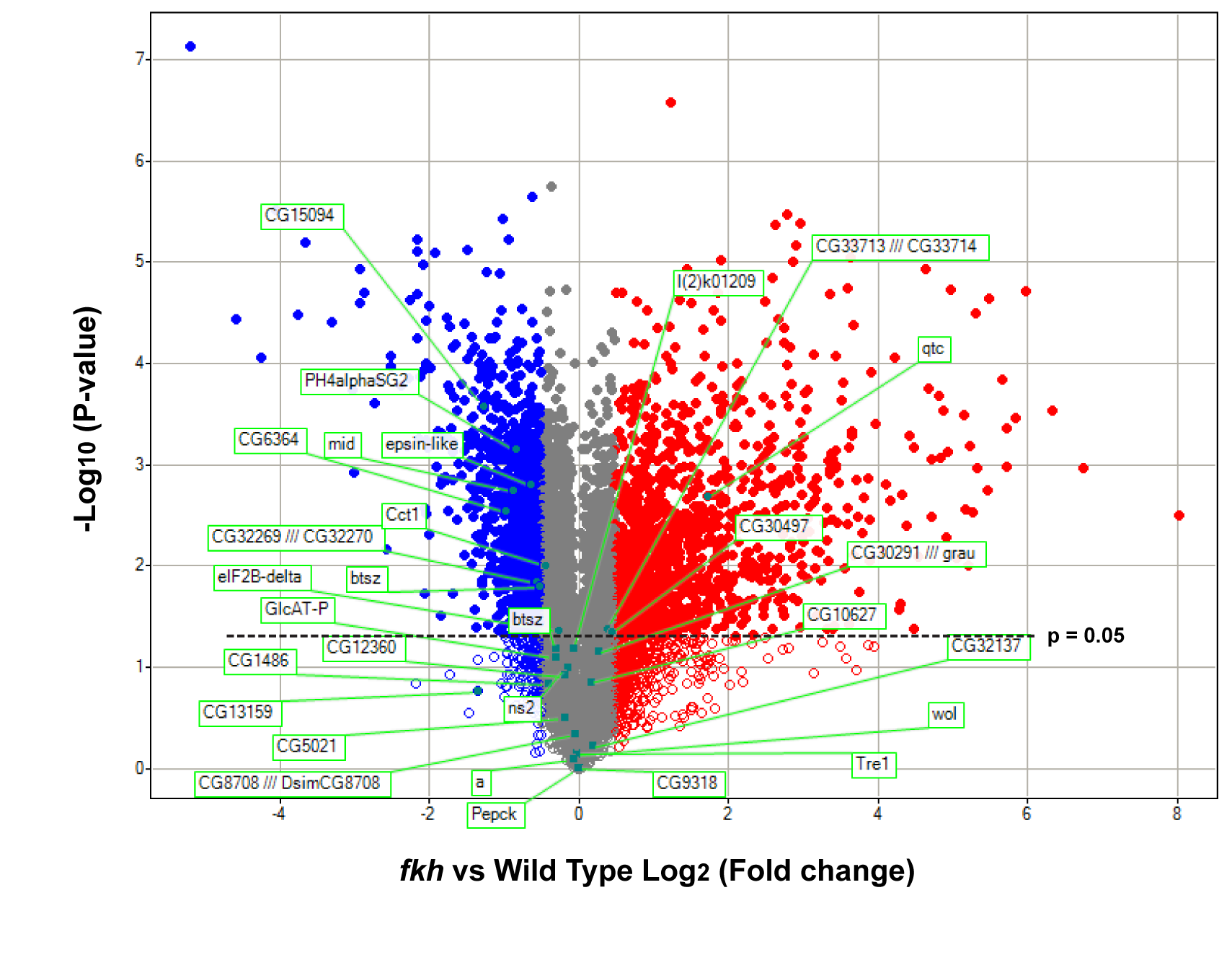

Supplement: Figure S1 — Volcano plot of gene expression changes between stage 11 WT and fkh mutant embryos. Genes whose expression is significantly downregulated in fkh mutants are shown with blue filled circles and genes whose expression is sifnificantly upregulated in fkh mutants are shown with red filled circles. Genes whose expression changed but with P-values greater than 0.05 are shown with open circles. Highlighted in green are Fkh-dependent SG genes identified by our in situ hybridization analysis. (TIF) [file pone.0020901.s001.tif]
